# Use of a laryngeal tube for sialolithotomy in a pediatric patient with left vocal cord paralysis: a case report

**DOI:** 10.1186/s40981-023-00629-5

**Published:** 2023-06-17

**Authors:** Masashi Fujii, Nobuaki Shime, Yasuyo Kawabata

**Affiliations:** 1 Department of Anaesthesia, Nagahama Red Cross Hospital, 14-7 Miyamae, Nagahama, Shiga 526-8585 Japan; 2 Department of Emergency and Critical Care Medicine, Graduate School of Biomedical & Health Sciences, Hiroshima University, Hiroshima, Japan

**Keywords:** Salivary gland, Calculi, Pediatric, Supraglottic, Airway device, General anesthesia

## Abstract

**Background:**

The laryngeal tube (LT), a closed esophageal supraglottic device comprising pharyngeal and esophageal cuffs, is used for emergency airway management. However, it is not often used in intraoperative airway management.

**Case presentation:**

A 9-year-old boy was scheduled for a sialolithotomy for sialolithiasis. He had a history of surgery for tetralogy of Fallot and had undergone vocal cord fusion for postoperative left-sided vocal cord paralysis. Following a strong request by the mother to avoid tracheal intubation to reduce the risk of bilateral vocal cord paralysis, management without intubation was initially considered in the preoperative anesthesia plan. Airway management was planned using an LT in case of ventilation failure due to positional abnormalities. Although some leakage was observed during intraoral surgery, it was quickly resolved by adjusting the position of the LT outside the sterile surgical field.

**Conclusions:**

The LT may be a viable option in cases where tracheal intubation is not preferred.

## Background

A laryngeal tube (LT) is a closed esophageal supraglottic airway management device comprising pharyngeal and esophageal cuffs. Because of the ease associated with its insertion and its minimally invasive nature, the LT is used for emergency airway management [1]. Products with a gastric tube lumen enable the insertion of a gastric tube and facilitate intragastric decompression [2]. However, they are not often used in intraoperative airway management.

Tracheal intubation should be avoided in patients with unilateral vocal cord paralysis, as they risk bilateral vocal cord paralysis. When using supraglottic instruments to secure the airway in intraoral surgery, there is a risk of dislocation of the supraglottic instruments during the surgery. There are reports of tonsillectomy and dental surgery managed with laryngeal mask airway (LMA) in pediatric patients, but none for sialolithiasis surgery. The risk of accidental dislocation of the supraglottic airway device associated with the surgical procedure may be particularly high in sialolithiasis surgery; this is because the visibility of the shaft of the airway device from the operative field is lesser during sialolithiasis surgery than during tonsillectomy or dental surgery. Furthermore, pediatric patients require rapid tube repositioning in the event of poor ventilation due to the rapid rate of decrease in oxygenation compared with that in adults. However, flexible tubes, such as the LMA Flexible™ Airway or Auraflex, are difficult to reposition from outside the sterile surgical field.

Here, we report the case of a pediatric patient with left vocal cord paralysis who was scheduled to undergo left sialolithiasis surgery.

## Case presentation

A 9-year-old boy (height, 125 cm; weight, 25 kg) was scheduled for a left sialolithotomy for sialolithiasis at a general hospital. He had a history of surgery for tetralogy of Fallot and had undergone vocal cord fusion for postoperative left-sided vocal cord paralysis. The preoperative examination revealed left vocal cord paralysis, although the blood test and cardiac function results were normal. His usual activities of daily living were unrestricted.

There was a strong request by the mother to avoid tracheal intubation to reduce the risk of bilateral vocal cord paralysis. Management without intubation was initially considered in the preoperative anesthesia plan. The surgery was to be performed orally, and the sublingual area needed to be deployed for surgical manipulation. Therefore, airway management was planned using an LT (VBM LTS-D, Medizintechnik GmbH, Sulz am Neckar, Germany), among other supraglottic airway-securing devices. Written informed consent was obtained from the mother to publish this report.

Slow induction of anesthesia was performed using 2 L/min of oxygen, 4 L/min of nitrous oxide, and 8% sevoflurane. After administering 20 mg of rocuronium and direct laryngoscopy using a laryngoscope, LTS#2 was inserted. After insertion, 30 mL of air was injected into the cuff. The shaft of the LT was moved to the side of the tongue to prevent interference with the tongue during surgery under direct laryngoscopy. Once confirmation was obtained that ventilation was possible, the pressure was reduced to 20 cmH_2_O using a cuff manometer. Intraoperative general anesthesia was maintained with 1 L/min oxygen, 2 L/min nitrous oxide, and 1.5% sevoflurane. Respiration was managed with a fraction of inspired oxygen of 0.4, peak inspiratory pressure of 15 cmH_2_O, respiratory rate of 18/min, and pressure-controlled ventilation with a single ventilation volume of 150–200 mL. Although some leakage was observed during intraoral surgery, the problem was easily solved by adjusting the position of the LT from outside the sterile surgical field.

The operation time was 12 min, the anesthesia time was 47 min, and the duration between LT insertion and LT removal was 26 min. After recovery from anesthesia, the patient showed no issues, including upper respiratory tract symptoms, and was subsequently transferred to the ward. The patient’s postoperative course was uneventful, and he was discharged the following day. No complications were noted at the follow-up a week later.

## Discussion

In this case, it was necessary to manage the airway using a supraglottic device deployed below the tongue. As an alternative to tracheal intubation, we also considered using an LMA, such as LMA Flexible™ Airway or Auraflex [3, 4]. These LMAs have a highly flexible tube and can be used for mouth angle fixation. In children, they have been used in head and neck surgeries, such as tonsillectomy, adenoidectomy, and dental surgery [5–7]. Conversely, during sialolithotomy, the surgical field must be secured by lifting the tongue. This requires the tube to be positioned more laterally than in tonsillectomy or dental surgery, potentially leading to inadequate ventilation.

Additionally, the shaft of the airway device is subjected to frictional forces that pull it out to deploy the lower part of the tongue. When excessive forces are exerted on the shaft, supraglottic devices with flexible tubes, such as LMA Flexible™ Airway or Auraflex, may dislodge from their position, even if they are secured at the mouth angle. In children, as observed in this case, the use of excessive force is even more dangerous because the tube is thin and soft. Furthermore, in the case of surgery for sialolithiasis, unlike dental surgery or tonsillectomy, the shaft of the airway device is obscured by the tongue and is challenging to visualize. Thus, monitoring and paying attention to its position can be arduous. We chose to use an LT in this case because we hypothesized that if the tube was mispositioned, it could be quickly readjusted by an anesthetist from outside the sterile surgical field.

To the best of our knowledge, there are no reports on the intraoperative airway management of patients with vocal cord paralysis using an LT, which is a closed esophageal supraglottic airway-securing device with a pharyngeal cuff and an esophageal cuff. The pharynx is closed with a large balloon; therefore, ventilation difficulties are unlikely, even if the tube is moved laterally. Compared to LMA, the maximum intraoperative ventilation pressure and complication rate associated with the use of an LT are the same [8]. The LT has a higher sealing power in the oropharynx than LMA Supreme, even in the neck extension position with the face turned right [9]. In out-of-hospital emergency airway provision, fewer positional abnormalities have been reported during resuscitation than tracheal intubation [10].

Patients can be ventilated at a lower cuff pressure using an LT than using LMA or Combitube [11]. In addition, balloon pressure should be adjusted to less than 60 mmH_2_O when using an LT [12]. In the present case, a cuff manometer reduced the pressure to less than 20 cmH_2_O. Additionally, the pharynx was examined using a laryngoscope during insertion to minimize the risk of damage to the vocal cords or mucosa.

In the present case, using an LT in a patient with salivary lithiasis helped avoid tracheal intubation and enabled the successful completion of the procedure without complications such as intraoperative airway problems.

There are some limitations associated with this report that need to be considered. In the present case, there were few problems due to the short duration of the surgery; however, problems may arise in the case of longer surgeries. As this is an exploratory case report, further cases are needed. Additionally, reports of the use of LT in children are still scarce, and it is not clear whether the results obtained for adults apply to children.

Using an LT in a pediatric patient with left vocal cord paralysis for sialolithiasis surgery allowed the avoidance and management of tracheal intubation. We suggest that the LT may be a practical choice as one of the devices to secure the supraglottic airway in cases where tracheal intubation is to be avoided, such as in patients with vocal cord paralysis.

## Data Availability

Data sharing does not apply to this article as no datasets were generated or analyzed during the current study.

## Abbreviations

LT: Laryngeal tube
LMA: Laryngeal mask airway

## References

[1] Dörges V, Ocker H, Wenzel V, Schmucker P (2000). The laryngeal tube: a new simple airway device. Anesth Analg.

[2] Dörges V, Ocker H, Wenzel V, Steinfath M, Gerlach K (2003). The laryngeal tube S: a modified simple airway device. Anesth Analg.

[3] LMA® Flexible™ Airway. Teleflex Medical Europe. https://www.lmaco.com/products/lma%C2%AE-flexible%E2%84%A2-airway. Accessed 22 Feb 2023.

[4] Ambu® AuraFlex™ disposable laryngeal mask. Ambu Inc. https://www.ambuusa.com/airway-management-and-anaesthesia/laryngeal-masks/product/ambu-auraflex-disposable-laryngeal-mask. Accessed 22 Feb 2023.

[5] Doksrød S, Løfgren B, Nordhammer A, Svendsen MV, Gisselsson L, Raeder J (2010). Reinforced laryngeal mask airway compared with endotracheal tube for adenotonsillectomies. Eur J Anaesthesiol.

[6] Alexander CA (1990). A modified Intavent laryngeal mask for ENT and dental anaesthesia. Anaesthesia.

[7] Gravningsbråten R, Nicklasson B, Raeder J (2009). Safety of laryngeal mask airway and short-stay practice in office-based adenotonsillectomy. Acta Anaesthesiol Scand.

[8] Somri M, Vaida S, Garcia Fornari G, Mendoza GR, Charco-Mora P, Hawash N (2016). A randomized prospective controlled trial comparing the laryngeal tube suction disposable and the supreme laryngeal mask airway: the influence of head and neck position on oropharyngeal seal pressure. BMC Anesthesiol.

[9] Wang HE, Schmicker RH, Daya MR, Stephens SW, Idris AH, Carlson JN (2018). Effect of a strategy of initial laryngeal tube insertion vs endotracheal intubation on 72-hour survival in adults with out-of-hospital cardiac arrest: a randomized clinical trial. JAMA.

[10] Yildiz TS, Solak M, Toker K (2007). Comparison of laryngeal tube with laryngeal mask airway in anaesthetized and paralysed patients. Eur J Anaesthesiol.

[11] Asai T, Kawashima A, Hidaka I, Kawachi S (2002). The laryngeal tube compared with the laryngeal mask: insertion, gas leak pressure and gastric insufflation. Br J Anaesth.

[12] Asai T, Shingu K (2005). The laryngeal tube. Br J Anaesth.

